# Challenges to fair decision-making processes in the context of health care services: a qualitative assessment from Tanzania

**DOI:** 10.1186/1475-9276-11-30

**Published:** 2012-06-07

**Authors:** Elizabeth H Shayo, Ole F Norheim, Leonard E G Mboera, Jens Byskov, Stephen Maluka, Peter Kamuzora, Astrid Blystad

**Affiliations:** 1 Department of Public Health and Primary Health Care, University of Bergen, P. O. Box 7804, 5020 Bergen, Norway; 2 Centre for International Health, University of Bergen, P.O. Box 7804, 5020 Bergen, Norway; 3 National Institute for Medical Research, P.O. Box 9653, Dar es Salaam, Tanzania; 4 DBL - Centre for Health Research and Development, Faculty of Life Sciences, University of Copenhagen, Thorvaldsensej 57, DK 1871 Frederiksberg, Denmark; 5 Institute of Development Studies, University of Dar es Salaam, P. O. Box 35169, Dar es Salaam, Tanzania

**Keywords:** Fairness, Decision-making processes, Health care services, Health systems

## Abstract

**Background:**

Fair processes in decision making need the involvement of stakeholders who can discuss issues and reach an agreement based on reasons that are justifiable and appropriate in meeting people’s needs. In Tanzania, the policy of decentralization and the health sector reform place an emphasis on community participation in making decisions in health care. However, aspects that can influence an individual’s opportunity to be listened to and to contribute to discussion have been researched to a very limited extent in low-income settings. The objective of this study was to explore challenges to fair decision-making processes in health care services with a special focus on the potential influence of gender, wealth, ethnicity and education. We draw on the principle of fairness as outlined in the deliberative democratic theory.

**Methods:**

The study was carried out in the Mbarali District of Tanzania. A qualitative study design was used. In-depth interviews and focus group discussion were conducted among members of the district health team, local government officials, health care providers and community members. Informal discussion on the topics was also of substantial value.

**Results:**

The study findings indicate a substantial influence of gender, wealth, ethnicity and education on health care decision-making processes. Men, wealthy individuals, members of strong ethnic groups and highly educated individuals had greater influence. Opinions varied among the study informants as to whether such differences should be considered fair. The differences in levels of influence emerged most clearly at the community level, and were largely perceived as legitimate.

**Conclusions:**

Existing challenges related to individuals’ influence of decision making processes in health care need to be addressed if greater participation is desired. There is a need for increased advocacy and a strengthening of responsive practices with an emphasis on the right of all individuals to participate in decision-making processes. This simultaneously implies an emphasis on assuring the distribution of information, training and education so that individuals can participate fully in informed decision making.

## Background

This paper focuses on decision making processes in health care with a particular emphasis on the potential influence of gender, wealth, ethnicity and education. Decision-making in health care is a complex process that ideally means identifying and choosing between alternatives on the basis of the values and preferences of the stakeholders in question. Fair process grounded in liberal democratic theory implies the involvement of stakeholders who discuss and reach an agreement based on reasons that are justifiable and appropriate in meeting people’s needs [1]. Stakeholders in health care include managers, care providers, patients, and the leaders and members of communities. The active participation of stakeholders in decision-making processes is one of the fundamental principles in primary health care (PHC) in the Alma-Ata Declaration[2]. It implies the delegation of power and the inclusion of all segments of the population to ensure that everyone gets an opportunity to participate effectively in decision making related to issues that affect their lives.

Active participation is achieved through a joint process of sharing ideas which enables individuals to influence decisions in a ‘representational’ manner [3]. The basic assumption is that shared decision making helps to improve the quality of the decision-making process, and, in the context of health, improves health outcomes [4]. This line of thinking is grounded in liberal democratic theory and decentralisation policy which argues for the importance of involving stakeholders in decision making processes. Immense challenges remain as to how to ensure stakeholder participation and how to decide at what points they should be actively involved.

The reasoning behind active participation in health care-related decision making moves beyond the equity aspect. Stakeholder involvement has been deemed vital in the sense that it enhances the likelihood that local needs are addressed hence increasing efficiency and responsiveness in health service delivery. A fundamental equity principle is that everyone affected by a particular decision is involved in the process with their ideas being listened to and taken into consideration. This approach is perceived to enhance the chance that individuals can access the basic needs necessary to protect and maintain good health [5]. It has been demonstrated that people prefer to implement ideas that they, themselves, find important [6] rather than those imposed by others [5]. Because of different values and interests among the stakeholders, deliberative democratic thinking puts an emphasis on deliberation and joint reflection [1]. Joint reflection is achieved through consensus building or through voting. However, a majority vote does not necessarily guarantee that the decisions made are the most appropriate ones. For a well functioning health system, empowerment of stakeholders [7,8] through awareness raising is important so they can be fully involved and can vote on the aspects they think are important to them. This will ultimately enhance fairness in decisions being made.

Deliberative democratic theory calls for collective decisions that are arrived at by stakeholders when they come together. Through deliberations, moral disagreement can be resolved with reasons that are justifiable by stakeholders who can think and act fairly despite the presence of different interests [1]. Deliberative democracy has been defined by Cohen as an association whose affairs are governed by the public deliberation of its members [9]. Gutmann and Thompson have suggested three ‘fundamental principles’ as keys in deliberative democracy theory; publicity, accountability and reciprocity [1]. Publicity in this context means that reasons behind decisions should be publicly available and accessible. Accountability implies that decision makers are held responsible for particular decisions in ways that discourage biases and fraud, and Reciprocity implies that procedures are followed during discussions to ensure that everyone maintains respect for and listens to each other’s ideas and views. With an emphasis on these principles, deliberation can be achieved despite disagreement among the group members. Gutmann and Thompson argue; "when citizens reason reciprocally, they seek fair terms of social cooperation for their own sake; they try to find mutually acceptable ways of resolving moral disagreements" Pg.2[1]. To allow this to happen, it is vital to create, an environment that allows participation to take place.

Under the decentralisation policy and health sector reforms initiated in the 1990s in Tanzania, decision-making processes in health care services were devolved to the local authorities at district level [10,11]. Substantial emphasis was placed on community participation and on securing health care decisions that emerge from the grass-roots level. A key policy element has been to ensure that the community is actively involved in identifying and prioritising between the problem areas they experience. This approach is to enhance the fairness and legitimacy of the decisions being made and links with what is advocated in deliberative democratic theory. However, studies from Tanzania indicate that, despite the well formulated policy intentions of the decentralisation and the health sector reforms, community views are rarely taken into consideration in district-level decision-making processes [12-14]. Top-down and authoritarian approaches prevail in that managers make decisions based on their own assumptions, knowledge and priorities.

Even when stakeholders are involved, the extent to which the ‘reciprocity’ principle works is unclear. The aspects that can affect the ability and opportunity for individuals or segments of the population to make a contribution to and be listened to during decision-making processes in health care have not been assessed sufficiently in low income settings. The objective of this study was to explore the challenges to achieving fairness in decision-making processes with special focus on the potential influence of gender, wealth, ethnicity and education.

### The Tanzanian health care system

Tanzania is located in East Africa, and is made up of 26 regions and 129 districts. The health care system is structured from the community to the national level, and each level plays a defined role. Health services are provided in a pyramidal structure starting from the dispensary as the lowest level via the health centres and the hospitals, with the larger referral hospitals at regional or national level at the top. Although most health services are provided by the government (64%), there is a long history of faith-based health services as well as an increasing number of private health institutions and organizations [15]. There is a private public partnership in the delivery of health services.

There is also a hierarchical structure in health care decision making at district level. The Council Health Management Team (CHMT) has the mandate to prepare the council health plan and to make health care decisions that are submitted to the district Full Council for discussion and approval. The role of the CHMT is to relate actively both ‘downwards’ and ‘upwards’ in the system. Diverse committees exist within the district, as well as at lower levels, and their role is to develop plans to be submitted to the CHMT [16,17]. Apart from the CHMT members, other important stakeholders in this system include the local government authority, the managers of health facilities, health facility committees, health boards, non- governmental organizations, private health service providers, and members of the community. According to the principles of the Tanzanian decentralisation policy, the discussion about health related priority setting and decision making is to start from the community and health facility levels where different committees exist. Decisions made at the local levels are later to be forwarded to the CHMT and eventually to the Full Council. The aim is to ensure that the decision making process is informed by the people in the district. It has, however, been documented that in actual practice this flow is not adhered to, as many of the committees within the district remain inactive [18].

Our study was carried out in the Mbarali District. The district is located in the south western part of Tanzania. The study is part of a larger EU funded project entitled, ‘Response to accountable priority setting for trust’ (REACT) which had its base in the same district between 2006 and 2011 [19]. REACT assessed approaches to improving fairness in priority setting within the health sector drawing upon the framework, ‘Accountability for Reasonableness (AFR). Our study does not actively draw upon the AFR framework, which is directly linked to the dynamics of priority setting processes, but uses the findings obtained within the REACT project that reflect general decision making processes in health care. As explained above, the paper draws upon a theoretical approach based within deliberate democratic thinking to frame the study and make sense of the findings.

## Methods

### Study site

In the 2002 National Population Census, the Mbarali District had a population of 234,101 (114,738 males and 119,363 females) with an estimated annual growth rate of 3%. The district has strong rural characteristics. The main ethnic groups are Sangu, Hehe, Bena, Sukuma, Maasai and Nyakyusa, with Sangu and Nyakyusa being the most numerous. A majority of the inhabitants depend on subsistence rice farming and livestock keeping as the main economic activities. The district is served by public and private health facilities including two hospitals, two health centres and 43 dispensaries. Figures from 2002 indicate that 46% and 5.2% of the adult population had primary and secondary education respectively [20].

### Study design

The study applied a qualitative design with in-depth interviews as the main data collection technique. A qualitative method was chosen in an attempt to gain a detailed and nuanced description of the experiences with health care decision making. This method allowed for the follow up of topics arising during the course of the interviews. In addition to the interviews, one focus group discussion was carried out with members of the Council Health Management Team (CHMT) in order to discuss the findings emerging from the interviews.

### Recruitment of the informants

A purposive sampling technique was employed to recruit the informants. The investigators, in collaboration with the Mbarali District Medical Officer (DMO), discussed and agreed upon the criteria for the selection of the informants. Participation in the decision making process was used as a main criteria in the recruitment process. A total of 33 informants were included in the study: 23 in the interviews and 10 in the focus group. In the interviews, 11 informants were recruited at district level, seven at health-facility level and five at community level. The focus group discussion comprised 10 members at district level. The district-level informants were key members of the CHMT and co-opted members such as the malaria focal person, the district AIDS coordinator, and the reproductive and child health coordinator. Other targeted informants included district officials and representatives of non-governmental organisations (NGOs). Facility-based informants included health workers, among them the managers’ (head of the health facilities). In order to gain an indication of how perceptions of the factors influencing decision making in health care at district and facility levels compared with views at the community level, a few knowledgeable individuals were recruited. At this level, literacy and being influential in their respective localities were the additional criteria.

### Data collection

Interview guides were developed for each subcategory of informants. The guides were aimed to measure the gaps in the Accountability For Reasonableness conditions which the REACT project aimed at. They were also designed in an open manner in an attempt to generate data related to the potential influence of gender, wealth, ethnicity and education on decision making in health care. Questions related to the representation of women or members of particular groups, to their particular roles, examples of their participation/nonparticipation were included in the guide. The focus group guide covered the same topics. The guides were developed by REACT’s qualitative team, and were later refined and translated into Kiswahili. Five of the authors of this paper speak Swahili, and all interviews and the focus group discussion were carried out in Swahili, which is the lingua franca of Tanzania. Although interview and topic guides were used during the interviews, the researchers encouraged the informants to reflect broadly on the topic and were sensitive to themes that arose in the course of the interviews and discussions. With consent from the study participants, digital recorders were used to record the interviews and the discussion. For those who did not wish to be recorded (five out of 23 in-depth interviews), detailed handwritten notes were taken by a research assistant. The notes were carefully reviewed and refined in detail immediately after the interviews took place. The interviews and the focus group discussion lasted between one and two hours each. Informal discussions with informants at district, health facility and community levels took place during the data collection period. These conversations contributed to deepening the understanding of the findings emerging from the interviews, and created grounds for further probing in particular areas. Handwritten field notes were made on a daily basis.

### Data analysis

The recorded interviews and focus group discussions were transcribed verbatim, and were later translated from Kiswahili to English with an emphasis on retaining culturally embedded expressions. After translation had been completed, the first author carefully read all the transcripts and notes and listened to all the recordings to get to know the full material well. Thereafter, a process of detailed coding was carried out manually drawing upon the pre-defined major categories of gender, wealth, ethnicity and education as a general guide. This enabled us to identify the specific pieces of text that expressed the informants’ experiences and perceptions related to the influence of gender, wealth, ethnicity and education on of decision-making processes in health care. Brief quotes or summaries of the content were noted in the margins of the transcripts. Recurring issues or patterns as well as nuances, ambiguities or contradictions within the emerging topics were systematically searched for. Information obtained from the interviews and the focus groups were triangulated in the analysis process to enhance the confidence of the data. The information gained during the informal conversations was also reviewed again at this point to increase the understanding of the material, but no direct quotes are drawn from the field notes in the results section. At each step the investigators discussed the emerging findings to enhance the soundness of the interpretation, with the first and last author being most active in the process.

### Ethics

The study received ethical approval from the Medical Research Coordinating Committee of the National Institute for Medical Research, Tanzania (NIMR/HQ/R.8a/Vol. 1X/416). Permission to conduct the study was further obtained from Mbeya regional and Mbarali district authorities. Permission to use the data was also obtained from the REACT scientific committee. The objective of the study was clearly expressed to informants before written informed consent was sought. The principles of voluntariness, rights of withdrawal, confidentiality and anonymity were strictly adhered to throughout the study.

## Results

Owing to the targeting of district officials and health workers, the informants were more educated than the average population. Only five out of the 23 informants in the interviews and two in the focus group were women. This gender bias was related to the fact that the levels from which our informants were recruited were dominated by men. The age range of the informants was 39–70 years: 40–54 at district level, 39–55 at health-facility level and 43–70 at community level. As explained above, informants at all levels were asked to reflect broadly on their knowledge and experience regarding the potential influence or lack of influence of the dimensions of gender, ethnicity, wealth and education on health related decision making processes. 

### Gender

Informants were asked about the level of women’s representation in and contribution to decision-making bodies, the extent to which their views were taken into consideration, how women’s participation was perceived and potential barriers to their participation and influence.

The initial response of the study informants at all levels referred to the clear political agenda of Tanzania, and emphasised the importance placed on gender considerations in decision-making bodies. It was reported that at governmental level it is spelt out clearly how many men and women are to be part of various committees. Decision-making bodies followed these guidelines so women were well represented. Women were said to be appointed to central positions, and informants said that women’s views were listened to and taken into consideration in the same way as those of men. They explained that what matters in decision-making processes is the strength of the arguments made and not the gender of the person raising the concern. The following statement was common throughout the interviews:

“Nowadays the gender issue is considered. Women are given leadership positions. In this district, the District Commissioner and Education and Agriculture Officers are women. We have a woman in the Council Health Management Team, and she is involved in everything at the office. If she is not present a meeting is postponed. Women are given opportunities to contribute and are listened to like men.” (District informant, male)

It was maintained by most of the informants that the more women gain confidence and influence, the more fairness will be achieved.

In the course of the interviews a far more nuanced picture of women’s actual involvement in decision making processes emerged. For example, informants explained that actual voice given to a woman depended on the section or committee in which she works. Women were said to have particular influence in the district meetings where decisions about maternity issues are discussed. Therefore the opinions of women were particularly listened to and valued in these sub-meetings and sub-committees. At the community level, women were also reported to be given substantial influence in the Village Health Committees, as they are the main implementers of health-related issues at a family level. It emerged that beyond the women- dominated spheres of maternal health, women’s attendance in and contribution to health related discussions were far from obvious. The discrepancy between the ideals of equality in terms of representation in diverse committees and the actual practice was also questioned during the interviews. One informant put it this way:

“If you think carefully about our district you will find that women make up the majority (of the population), but they are the minority in the decision-making bodies. In the district council, we have three women out of the total of 11 councillors. Now, when voting, even if they (the female representatives) have an important issue to bring up, when counting the votes they lose. I will say that women aren’t sufficiently represented, and I think this is not solely this district’s problem but a problem found in the entire country.” (District informant, male)"

Informants held that the fewer of females in the decision making bodies, the smaller the chance for their views to be taken on board because, they would be outvoted.

Many male informants claimed that low levels of education were a challenge for women’s involvement in the decision making bodies. The necessary expertise among women was often lacking. Informants said that, even when vacancies were advertised, women would not apply for the positions because they lacked formal skills, and they could not be forced to apply. This challenge was related to a lack of adequate skills, interest and ability. This scenario was said to make it difficult to implement the official guidelines of equal representation of men and women in decision-making bodies at the district level.

In the course of the interviews, differences emerged between male and female informants regarding women’s influence. Male informants emphasised women’s participation more strongly while female informants brought up numerous complaints related to women’s actual roles in decision-making bodies from the community to the district level. Female informants argued that women’s views were not sufficiently listened to.

“An opinion can be rejected just because it comes from a female member… A woman can argue for the importance of providing training related to health service provision in the planning meetings, but the issue may not be considered, as other suggestions like constructing buildings (suggested by men) are given priority.” (District informant, female)"

Women were said to end up crying sometimes because of frustrations resulting from being undermined by men as revealed in the following quote;

“My opinions are taken into account because of my confidence and standing. But sometimes women are even crying in the planning meeting as their views are not taken into consideration” (District informant, female)"

It was held that, even for the educated women, it was difficult to get their views through simply because they came from women. At the community level, the challenges of women’s involvement in decision making emerged as particularly serious with more direct reference to the female gender *per se*. One female community informant explained:

“In the meetings, even when there are knowledgeable women present, we are not listened to when we present our views. We are always asked, "Who are you?”"

Throughout the interviews at all levels it was maintained that women had not gained sufficient confidence in formulating and presenting ‘strong points’ (hoja za msingi). It was held that little public exposure and shyness made them lag behind. Apart from reservations regarding women’s skills and competence, a scepticism regarding the appropriateness of women’s involvement in decision-making bodies emerged, particularly at community level. A lack of trust in women’s abilities to carry out proper assessment and decision making emerged among some of the informants. They were very direct in expressing their views about women’s incompetence as one said:

“There are very few things which women can do because of their nature. There are things which we just force them to do, although we know that they really can’t do them. For example women cannot supervise the construction of the dispensary, so why should we listen to their opinions?” (Community informant, male)"

It was concluded by the majority of the informants that the actual influence of women varies starkly from one decision-making body to another and from one level of authority to another, with the community level facing the greatest challenges in terms of ensuring the inclusion of women’s views.

### Wealth

The potential impact of economic status on decision-making processes in health care services was also explored. At the district and health facility levels, a very limited influence of wealth was recorded from the informant’s statements. Informants stated that in areas where guidelines were properly followed, the influence of well-to-do people was minimal. One informant concluded:

“We are not influenced by an individual’s economic status. If you are well-off, it is relevant to you and your family but not for the hospital management team. What matters here is how strong a person’s arguments are.” (Health facility informant, male)"

Informants also argued that at the community level the wealth of a person had little impact, particularly if it was combined with low education. One informant stated:

“… People are after constructive ideas and only that. What is more, rich people are few in our village. Others may have many cattle, but they don’t have a substantial influence owing to their poor education.” (Community informant, male)"

A different picture of the influence of wealth emerged as we moved closer to the community. Informants acknowledged that as far back as people could recall the wealth of a person has influenced decision-making processes. A continued impact of rich people emerged because of their ability to offer assistance in various matters. Rich individuals used their power to influence decisions in more direct ways, as this quote illustrates:

“There are individuals here known as "Burushi". These people are a mixture of Arabs and Africans, and are financially well-off. They have plenty of money. In the meetings, if they want a certain decision to be made, even if it is of no benefit to the community, it is commonly accepted. Decision makers have no choice as the "Burushi" make substantial contributions to health-related issues.” (District informant, male)"

A more common phenomenon touched upon by almost all the informants was the ability of rich people to influence decision-making processes more directly through bribery. Bribery brings wealth to the heart of decision making, and gives affluent individuals more power. The asset implied by the well-to-do was linked with male gender.

It emerged in the interviews that when a wealthy person speaks he is listened to more than others, not only because he is in a better position because of his resources, but because of perceptions that wealthier people are more ‘intelligent’ than the poor. One informant put it in the following way:

“I must be frank; a poor person’s influence on the decision-making process is minimal because of his status. He might have good ideas, but because he is poor, he has no influence… It is the opinions of the rich person that to a large extent are implemented. I know myself that when you have a good life you also have a good ability to think, but if you are poor your thinking capacity becomes limited as you are thinking about very small things. While you are thinking about stiff porridge (ugali) others are thinking of cars. Therefore, to convince people becomes really hard because you’re thinking: "How am I going to get my lunch today?" while your friend is thinking of cars and machines.” (Community informant, male)"

The manner in which a degree of legitimacy was given to such scenarios emerged in several of the interviews and informal talks at community level. Moreover, the influence of wealth emerged as more pronounced when it was coupled with high education of an individual.

### Ethnicity

As with the other points raised, the immediate response from the district and health facility informants regarding the potential influence of ethnic affiliation was that ethnicity had a very limited influence in Tanzania. This instant reply was situated within the discourse of the late Tanzanian President Julius Nyerere, who used his entire career to advocate against differentiation based on ethnic criteria. The fact that members of the district decision-making bodies and facility committees would always belong to different ethnic groups was also brought up as a factor that worked against tribalism. It was maintained that individuals in such positions were obliged to follow governmental rules and regulations, which makes it very difficult to promote decisions that favour particular ethnic groups. Emphasis was again placed on an individual’s knowledge and skills relating to a particular topic. One informant stated:

“… Although you may find that a majority of the health staff in a certain unit/department originates from the same ethnic group, when it comes to decision making in health care services, the person’s capabilities or skills are considered to a greater extent than their ethnic affiliation.” (District informant, male)"

Regarding gender and wealth, a more complex picture did emerge in the course of the interviews. For example, informants expressed that a leader at any level will listen far more attentively to the opinions from individuals who originate from his/her own ethnic group. District and health facility informants provided numerous examples of how ethnic affiliation was made relevant concerning issues such as staff transfers, payment of allowances, promotions and training opportunities. The following quote illustrates:

“… Here there is a department that is dominated by a certain ethnic group. When it comes to decision making, you may reach an agreement in relation to a particular health issue, but later you find that the decision has been changed without any official reason. If you ask yourself who changed the decision, you will realise that it is the head of the department, who originates from the same ethnic group as the person who ends up being favoured. He commonly favours his "colleagues" (from the same ethnic group) and that is not a secret here.” (District informant, female)"

Informants expressed the view that, in the community, certain ethnic groups have a strong tendency to dominate or influence others. The largest ethnic group in the community was said to use their numerical advantage to exploit or oppress others. This was even more apparent as this group was also wealthy. Large and strong clans within particular ethnic groups could also have undue influence as reflected in the following quote:

“… There is a certain clan in this community with a very strong influence in decision-making meetings. Even the local government leaders are afraid of them. It is a very big clan that affects the government of this village. This is also a rich clan that doesn’t follow government regulations.” (Community informant, female)"

### Education

Informants perceived educational level as a very important factor in decision-making processes. Educated individuals and professionals were strongly depended upon by their leaders when making decisions. The importance of education was emphasised strongly to an extent where local knowledge was devalued. One informant said:

“… An educated person first of all is a professional and the advice he gives has scope. Opinions and decisions given by non-educated individuals are doubtful” (Community informant, male)"

Two different concerns regarding the representation of groups with low education emerged: one related to a lack of influence and the other related to too much influence in the decision making processes. It was argued that if individuals with lower level of formal education were better represented, common people’s problems would be more readily identified and addressed. One informant had this to say:

“We, health facility managers are not involved in the District Health Committee meetings. As a result we don’t receive most of the things we are in need of. Let them invite us to these meetings even once a year, even if it will be at our own expense. They are afraid to call us because they fear being asked questions specifically related to the expenditures.” (Health facility informant, male)"

It was argued that a real challenge was linked to the fact that when the formal educational level of an individual is low, a person tends to lack the necessary confidence to take an active part in discussions, and will not be able to present his/her argument clearly. This view supported the argument provided by some informants that the attendance and representation of less educated individuals often does not lead to the desired results in terms of a true grass-roots engagement and does not impact on the decision-making processes.

There was a strong focus among the district level informants on the substantial influence of individuals with very low levels of education in the decision making processes. They referred specifically to the Full Council where the majority of the members are made up by the Councillors who represent communities. The informants from district level raised serious concerns that these councillors are given substantial power to engage in district-level decision making but often lack the education and expertise related to the issues they discuss and eventually vote on. It was held that as councillors, they are often not in a position to be well enough informed and to judge the issue at stake from different positions. According to the informants, the result are uninformed decisions;

“Most of the Councillors have little knowledge to conceptualise what is being discussed. Usually they attend the meeting just to listen, and when it reaches the time for voting they just agree and sign in order to pass the resolution. From my experience I can say that some of these members do not understand what is being discussed. Most of the health issues are not understood by non-medical personnel. A resolution may be passed with the understanding that the council has reached consensus, but in reality it might be a decision proposed and enforced by a single member of the council as the other voters simply have agreed but may not have understood the issue being discussed.” (District informant, male)"

The informants reported that the impact of grass-roots representation in actual practice was limited as the representatives were unable to grasp many of the issues at stake, and would vote in ways that would not favour community opinion. However, many informants would also argue that there are community members who, despite a lack of formal education, have an excellent ability to provide constructive ideas by drawing upon their varied competence and experience. Thus, a complexity of views were raised regarding the challenges of ensuring informed grass-roots engagement and grass-roots impact at a time when formal education and specialised knowledge is increasingly demanded.

## Discussion

This study indicates extensive limitations in terms of fair participation in the decision- making processes in health care in the study district in Tanzania. The influence of gender, wealth, ethnicity and education presents substantial challenges. At a general level, the tendency was clearly one of placing more trust and power in men, in wealthy and formally educated individuals as well as in individuals from powerful ethnic groups. The influence was more pronounced at the community level than at the district and facility levels. At the district level, the influence, particularly of wealth and ethnicity, was deemed to be minimal. This was attributed to the fact that members of decision making bodies would come from different ethnic origins and would have different economic status. For example in the Full Council meetings, these factors were said to hardly play a role as the members are obliged to adhere to government rules and regulations that strictly stipulate the procedures to be followed and it is not easy to deviate from them.

At the onset of the discussion, it is interesting to note the way the interviewees started their responses by addressing the importance of the principles of fairness in terms of gender, ethnicity, wealth and education, and the lack of discrimination on the basis of such characteristics. This immediate response was seen to become more nuanced and ambiguous in the course of the discussions. It is important to comment upon this seemingly ‘politically correct’ response with a brief reference to Tanzanian history.

In Tanzania, the former president Mwalimu J. K. Nyerere’s political agenda from independence focused on fighting against a class society based on poverty, disease and ignorance, which he saw as the main enemies of development. He worked on the basis of socialist ideals and the village became the core of his policy through the ‘ujamaa na kujitegemea’ (socialism and self reliance). A prime legacy of Nyerere was to unite all ethnic groups in the country through a joint language ‘Kiswahili’ [21]. Through this agenda, the battle against tribalism in Tanzania was fought through slogans such as ‘united we stand, divided we fall’ (‘umoja ni nguvu, utengano ni udhaifu’). This made Tanzania a showcase for maintaining peace and unity in a multi-ethnic setting [22]. Nyerere based his policy on social justice principles where each individual was to have the right to be respected and be listened to regardless of social status [23]. Despite his good intention, he did not spell out clearly the manner in which the grassroots’ level was to be heard in the face of a strong and authoritarian state.

The immediate response of the informants regarding gender, wealth, ethnicity and education must also be understood in light of the later health sector reform and the decentralisation policy in Tanzania which strongly advocate bottom-up approaches in decision making [24]. Emphasis is placed on community or grass root involvement where every individual is to participate equally in discussing their problem areas and suggesting solutions. The recognition and emphasis of the village or the community as the focus of or basis for development has remained central for close to 50 years in Tanzania. In recent years, active engagement of people in debate has been encouraged [24,25]. This has increased recognition of the importance of poor and marginalized segments of society having a right to air their grievances. The fundamental assumption is that when diverse stakeholders from grassroots are involved, decision making improves as it takes place closer to where the problems are located. Communities are called upon to take an active part in and to challenge decisions that affect their health. These visions have been a central part of Tanzania’s independent history, and it is within this contextual backdrop that the immediate response from the informants must be understood. However, the implementation of ideas of decentralization policy has largely remained theory [14]. The initial responses are also in line with the deliberative democratic thinking that in a fair process, there should be reasoning among equal citizens and shared commitment to the resolution. To achieve this, in the deliberation, stakeholders should decide on the agenda, discuss the issue, propose solutions and support those solutions with reasons [9]. Each stakeholder is to have equal voice in the decision making since the distribution of power and resources is not supposed to shape their chances of contributing or playing an authoritative role in the deliberations.

Beyond the initial response, our study findings indicate that gender, ethnicity, wealth and education do, in practice, pose substantial challenges in making fair decisions. This study cannot, in any substantial manner, quantify or explain the discrepancy between the levels of ideals and values on the one hand and the level of practice on the other. But we can indicate a few aspects of the challenges that emerged in our study findings, and ways in which some of these seem to not only appear at the level of discriminatory practice, but also at the level of ideas and ideals in a way that may impose serious constraints on principles of fairness.

Our findings indicate that, despite the strong focus on gender balance in decision-making bodies, substantial challenges remain. There is still a lack of women with the necessary formal competence or skills to occupy certain positions. Beyond this, women were said to be listened to less seriously during discussions than their male counter parts. Informants expressed the view that, in meetings where educated and active women were involved, it was often difficult for them to be heard beyond the field of maternal and child health. At the community level, the findings were even more serious as the fundamental ability of women to make a meaningful contribution to the discussions was questioned by several of the male informants, revealing a true distrust between the genders. This lack of ability was not merely linked to a lack of experience in voicing their views or to a lack of formal education or training, but was related to their nature as women. These findings reflect strong traces of patriarchal ideology as have been found also in a number of other studies [26-28].

A similar line of reasoning emerged from the findings related to wealth and influence. People with higher income were reported to be listened to more than the poor. This finding emerged as far more apparent at lower levels, and not least at the community level. Informants at the community level argued that the rich would be in a better position not only because of their financial resources, but because poor individuals were perceived to have lower thinking capacity; the more affluent were perceived to be more intelligent than the poorer. The influence of wealth has been reported in another study where members of decision making bodies were chosen because of their fundraising ability [29]. This implies that poor people’s views will be heard to less extent, although they may be the ones who may experience a problem more acutely and may be most affected by the potential decisions. This tendency to allow the wealthy to have more influence has been pointed out as reason for caution also in other studies [30] if fairness is to be achieved. Despite the enormous historical focus on the dangers of tribalism, ethnicity did also emerge as challenge in our study. The majority ethnic group in the community was said to be more likely to be respected and listened to than other groups, not least if its power in terms of numbers was coupled with wealth. A bias was noted also in the district departments when a majority of the staff belonged to a particular ethnic group. Our findings indicate that advantage or disadvantage based on ethnic criteria in decision-making contexts needs to be watched carefully also in present day Tanzania.

The influence of education was, not surprisingly, pronounced. People with formal education were said to have substantial influence owing to their increased knowledge and competence, while individuals with little education had less influence. Other studies have found that educated individuals were thought to have more confidence [31] and thus feel more comfortable in engaging in complex discussion. In our study, the elected councillors who approve district decisions were considered by some informants to lack the necessary education and understanding to vote in an informed way in many of the questions addressed. These councillors have authoritative power, yet at times lack the necessary knowledge in approving decisions in health care. This point has been raised also in other studies [29,32-34]. With this in mind, the approaches to ensuring proper community representation need to be thought out, and the necessary knowledge and information need to be imparted to councillors so that they can make informed decisions for better health outcomes.

The study findings indicate that deliberative democratic thinking that advocates fairness and legitimacy in decision-making processes [1,9] continues to be undermined, and that it is not yet adequately practiced or conceptualised in our study district. The objective of participation in decision making is to make sure that decisions reached are informed by the people. For this principle to function, as the reciprocity principle of deliberative democratic theory states, each stakeholder needs to have an equal chance to contribute and being heard regardless of inherent power differences. Our findings bring a serious dilemma. How can one possibly take on board ideas from all stakeholders in an effort to enhance the democratic process and, at the same time be able to address the needs in an adequate and informed way as perceived by stakeholders themselves? Our study indicates that the basis for fair and legitimate decision making is far from being reached, and that the challenges need serious and renewed efforts. Despite this, enhancing fairness and legitimacy through the inclusion of people beyond powerful individuals is deemed vital, as shown by Kapiriri and Martin [35].

When informants question women’s or poor people’s innate ability to take part in informed decision making constructively, and consider it fair that other individuals legitimately act or decide on their behalf, we are not talking merely of discrimination, but of challenges to human rights-based fairness principles in a more fundamental way.

Prevailing biases affect people’s self esteem and sense of worth, which in turn affect their ability to be open, creative and vocal. On such grounds Gibson et al. propose adding ‘empowerment’ to the fairness conditions as proposed by the ethical framework Accountability for Reasonableness [36]. This line of thinking, emphasizes appropriate training and orientation to enable stakeholders to contribute substantially [14,33,37-39]. Empowerment has been defined as the process and outcome whereby those without power gain information, skills, and confidence and thus control over decisions pertaining to their own lives [40]. Empowerment processes can take place at the individual, organizational or community levels. Green argues; “The poor, divorced from centres of decision making dominated by elites with different interests, must be empowered to participate in the decisions which affect them” [7]. In a decision-making context, stakeholders should be obliged to respect the opinions of each other. This is the fundamental argument of deliberative theorists who advocate for mechanisms that reduce the influence of all asymmetric power relations and authoritarian approaches in decision-making processes. Deliberative democracy advocates for a just society where decisions are made collectively and become a public good [9]. Rawls clarifies that power in decision making has to be located independently of the economic and social position of individuals [41]. More consultative and participatory approaches are called for in an attempt to secure the participation of broader segments of the population [1,6]. The struggle to find ways to include the views of women, the poor, individuals from every ethnic segment and from both educated and non-educated parts of the population has to remain in focus in the years ahead.

### Strengths and limitations of the study

The findings of this study are based on a limited number of informants located at different levels within the district. There is nonetheless reason to believe that the findings have relevance beyond the study district as policies, bureaucratic structures and multi-ethnic environments are found in all parts of Tanzania. It is indeed likely that the findings may have relevance for many other settings in newly developing democracies where there has been less focus on community voice and involvement than in Tanzania.

## Conclusion

The findings from this study have revealed that fairness principles in health care decision making processes are greatly undermined in the present study district in Tanzania. Women, poor individuals, members of minority ethnic groups and less educated individuals were found to be discriminated against in decision-making bodies. The findings were more pronounced at community than at health facility and district levels. The findings revealed that such biases were related to perceptions of women, the less educated and poor individuals as less knowledgeable and having a lower thinking capacity. These notions imply fundamental challenges to the implementation of democratic and justice theories as spelled out by deliberative democratic thinking. We argue that such notions pose a very real threat in health care decision making as they may systematically undermine the views and experiences of particular segments of the population. There seems to be a prevailing lack of knowledge and also a lack of acceptance of the principles on which the political system is built, including the fundamental right of everyone to be heard. Intensive advocacy related to fairness principles and to people’s rights to participation in decision making processes should be strongly emphasised in the years to come. The clear distinctions between the findings at community levels and at district levels indicate that ensuring equal opportunities in terms of access to education and information will, in the long run, lead to a situation where stakeholders at every level are given a chance to participate in a fair way and make legitimate decisions in health care since they will be knowledgeable on the issues at stake. Only in this way can the true community voice be secured regardless of gender, wealth, ethnic origin and educational level.

## Competing interests

The authors declare that they have no competing interests.

## Authors’ contributions

EHS participated in the development of the tools, collected and refined the data, carried out the analysis and drafted the manuscript. AB was central in the process of developing the guides, made a follow-up visit to the field site during data collection, took part in the analysis process and revised the draft manuscripts. PK participated in the development of the tools, took part in the data collection process and reviewed the manuscript. OFN, LEGM and SM reviewed the manuscript several times. JB conceived the idea of the project, developed the methodology and reviewed the manuscript. All authors read and approved the final manuscript.
